# Immunohistochemical staining of radixin and moesin in prostatic adenocarcinoma

**DOI:** 10.1186/1472-6890-11-1

**Published:** 2011-01-14

**Authors:** Tanner L Bartholow, Uma R Chandran, Michael J Becich, Anil V Parwani

**Affiliations:** 1 University of Pittsburgh School of Medicine, Pittsburgh, PA, USA; 2 Department of Biomedical Informatics, University of Pittsburgh School of Medicine, Pittsburgh, PA, USA; 3 Department of Pathology, University of Pittsburgh School of Medicine, Pittsburgh, PA, USA

## Abstract

**Background:**

Some members of the Protein 4.1 superfamily are believed to be involved in cell proliferation and growth, or in the regulation of these processes. While the expression levels of two members of this family, radixin and moesin, have been studied in many tumor types, to our knowledge they have not been investigated in prostate cancer.

**Methods:**

Tissue microarrays were immunohistochemically stained for either radixin or moesin, with the staining intensities subsequently quantified and statistically analyzed using One-Way ANOVA or nonparametric equivalent with subsequent Student-Newman-Keuls tests for multiple comparisons. There were 11 cases of normal donor prostates (NDP), 14 cases of benign prostatic hyperplasia (BPH), 23 cases of high-grade prostatic intraepithelial neoplasia (HGPIN), 88 cases of prostatic adenocarcinoma (PCa), and 25 cases of normal tissue adjacent to adenocarcinoma (NAC) analyzed in the microarrays.

**Results:**

NDP, BPH, and HGPIN had higher absolute staining scores for radixin than PCa and NAC, but with a significant difference observed between only HGPIN and PCa (p = < 0.001) and HGPIN and NAC (p = 0.001). In the moesin-stained specimens, PCa, NAC, HGPIN, and BPH all received absolute higher staining scores than NDP, but the differences were not significant. Stage 4 moesin-stained PCa had a significantly reduced staining intensity compared to Stage 2 (p = 0.003).

**Conclusions:**

To our knowledge, these studies represent the first reports on the expression profiles of radixin and moesin in prostatic adenocarcinoma. The current study has shown that there were statistically significant differences observed between HGPIN and PCa and HGPIN and NAC in terms of radixin expression. The differences in the moesin profiles by tissue type were not statistically significant. Additional larger studies with these markers may further elucidate their potential roles in prostatic neoplasia progression.

## Background

Prostate adenocarcinoma is the second most commonly diagnosed cancer in American males, trailing only skin malignancies. During 2010 alone, it is projected that 217,730 new cases of prostate adenocarcinoma will be diagnosed in the United States. During the same year, 32,050 deaths attributable to this cancer are expected to occur [1]. Understanding the cellular protein expression patterns associated with prostate adenocarcinoma may provide greater insight into the processes of prostatic neoplastic growth and dissemination, as well as allow for the identification of novel prognostic biomarkers and therapeutic targets.

The Protein 4.1 superfamily is one of current interest in this regard. Many of this family's proteins serve to cross-link components of the cellular plasma membrane to the internal cytoskeleton [2,3], and have therefore been postulated to play a role in the processes of cellular adhesion [2,4] and, in some instances, growth and proliferation and the regulation of these processes [3,5,6]. Strong cytoplasmic immunohistochemical staining for ezrin, a member of the ezrin-radixin-moesin (ERM) subfamily of Protein 4.1 [6], has been associated with decreased survival in upper aerodigestive tract squamous cell carcinoma [7]. Additionally, ezrin positivity has been associated with decreased survival rates and incidence-free periods in hepatocellular carcinoma [8]. In prostatic adenocarcinoma specifically, its expression has been correlated with decreased tumor differentiation [9].

Conversely, loss of Protein 4.1B, another member of the same superfamily [3], has been shown to lead to an enhanced metastatic capacity during the induction of human adenocarcinoma PC-3 cells into immunodeficient mice [10]. These studies support the conclusion that not all members of this superfamily have the same effects on carcinogenic processes.

Two members of this superfamily whose prostatic tissue expression has not been well characterized in the literature are radixin and moesin. Radixin is an ~80 kDa protein [11] that has been shown to be down regulated in some cases of lung adenocarcinoma [12]. Additionally, its chromosomal location, 11q23, has shown a loss of heterozygosity in select incidences of lung, breast, ovarian, and colon cancer [13]. Moesin, a 78kDa protein [14], has been associated with decreased survival in oral squamous cell carcinoma when a cytoplasmic distribution pattern was observed [15]. Moesin-positive cases of pancreatic adenocarcinoma have been associated with shorter survival times than moesin-negative cases [16], with moesin-positive tumors demonstrating higher histopathologic grades and perineural and lymphovascular invasion rates [17]. Despite this, it, like radixin, has been shown to be down regulated in select cases of lung adenocarcinoma [12].

In this study, the immunohistochemical staining intensity of both radixin and moesin was examined in tissue microarrays of normal donor prostates (NDP), benign prostatic hyperplasia (BPH), high-grade prostatic intraepithelial neoplasia (HGPIN), prostatic adenocarcinoma (PCa), and normal tissue adjacent to prostatic adenocarcinoma (NAC). Low grade prostatic intraepithelial neoplasia was not studied both because the diagnosis is subjective and because it lacks clinical relevance. In general, the percentage of cases of HGPIN that feature prostatic adenocarcinoma on rebiopsy is 30% [18]. No specimens of HGPIN included in this study were diagnosed at the time as containing PCa.

## Methods

### Preparation of Tissue Microarray Blocks

Two sets of tissue microarray (TMA) blocks were constructed using specimens located in the Health Sciences Tissue Bank at the University of Pittsburgh Medical Center. All specimens were originally obtained through either a radical prostatectomy, transurethral resection of the prostate, or via a needle biopsy of the prostate, with the majority obtained using the first two methods. Cores were taken from the appropriate case specific paraffin-embedded tissue blocks and assembled into new TMAs as previously described [19]. Due to variations in TMA processing, 11 cases of NDP, 14 cases of BPH, 23 cases of HGPIN, 87 cases of PCa, and 24 cases of NAC were analyzed for radixin staining intensity. Eleven cases of NDP, 12 cases of BPH, 23 cases of HGPIN, 88 cases of PCa, and 25 cases of NAC were analyzed for moesin staining intensity. While every effort was made to include all of the cases in this study, processing artifacts within some of the TMAs cores made them unscorable, hence resulting in a difference in the numbers of cases between the two stains. For each set, at least four cores were taken from each case to ensure adequate sampling. Each case was included only if at least three cores were processed completely enough to be scored.

### Immunohistochemistry

Each TMA block was deparaffinized and subsequently rehydrated with incremental ethanol concentrations. Decloaker was then used for heat induced epitope retrieval, followed by a 5 minute TBS buffer rinse. The slides were then placed in a Dako Autostainer. One set of TMAs was stained with anti-radixin C-15 (working dilution 1:200), a polyclonal goat antibody (Catalogue # sc-6408) from Santa Cruz Biotechnology (Santa Cruz, CA, USA). The other set was stained with primary anti-moesin C-15 (working dilution 1:50), a polyclonal goat antibody (Catalogue # sc-6410) from Santa Cruz Biotechnology (Santa Cruz, CA, USA). Immunolabeling was conducted using Vectastain Elite Goat IgG -ABC Kit (Avidin/Biotin) from Vector Laboratories (Burlingame, CA, USA). Slides were counterstained with hematoxylin before being coverslipped.

### Scoring of Slides

Staining for both radixin and moesin was assessed in the cytoplasm of the cells of the prostatic glandular epithelium. Slides were scored on a scale of staining intensity, with 0 representing no staining, 1 representing weak staining, 2 representing moderate staining, and 3 representing strong staining. The intensity was then summed with the percentage of the core stained multiplied by four. In cores where more than one staining intensity was significantly represented, the average score for the core was calculated. This scoring procedure is adapted from a scoring protocol previously used by Parwani, et al. [20] The mean score for each case was then determined. Finally, the mean scores were obtained for each type of prostatic tissue represented. Means by Gleason score and tumor stage, where available, were also obtained. The Clinical TNM, as opposed to the Pathologic TNM, staging classification was used to assess the specimens. All means were reported with standard error.

One Way ANOVA analysis or Kruskal-Wallis tests were used to analyze the groups (α = 0.05). Two different statistical tests were used in this study, depending on whether the data in specific comparison met the statistical assumptions of being normally distributed and having equal variances between the groups, both necessary to perform a parametric analysis. If not, this necessitated a non-parametric Kruskal-Wallis test. Subsequent Student-Newman-Keuls tests for multiple comparisons were conducted to analyze differences (α = 0.05).

Photomicrographs of tissue cores were taken using an Olympus BX51 microscope using Spot Advanced V4.6 (Diagnostic Instruments, Inc.) software. All images were taken at 10x.This study received exempt approval (PRO08040368) from the University of Pittsburgh Institutional Review Board. This approval and abiding by the guidelines for usage of specimens from the Health Sciences Tissue Bank at the University of Pittsburgh permitted the use of all specimens included in this study.

## Results

### Age Classification

The average ages and standard deviations of the patients whose specimens were included in the TMAs at the time of specimen retrieval were 66.7 ± 6.2 for PCa, 66.9 ± 8.31 for BPH, 32.1 ± 12.7 for NDP, 67.9 ± 5.25 for NAC, and 66.5 ± 5.7 for HGPIN.

### Staining Intensities for Radixin

**Figure 1:**
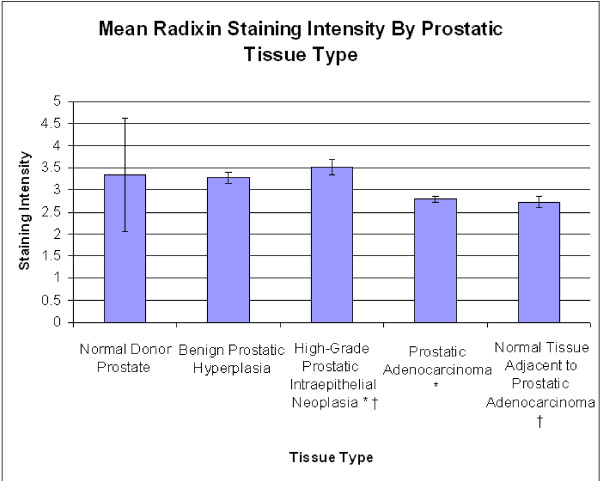
**Mean radixin staining intensity by prostatic tissue type**. Significant differences are seen between prostatic intraepithelial neoplasia and prostatic adenocarcinoma (p = < 0.001) and normal tissue adjacent to prostatic adenocarcinoma (p = 0.001). The asterisk (*) and the dagger (†) are used to signify the tissue types that are significantly different.

The mean staining scores for NDP, BPH, HGPIN, PCa, and NAC in the radixin-stained TMAs were 3.34 ± 1.29, 3.27 ± 0.12, 3.51 ± 0.18, 2.79 ± 0.08, 2.72 ± 0.13 (Figure 1). A One-Way ANOVA (p = < 0.001), with subsequent Student-Newman-Keuls tests for multiple comparisons, showed significant differences between HGPIN and NAC (p = 0.001) and HGPIN and PCa (p = < 0.001).

**Figure 2:**
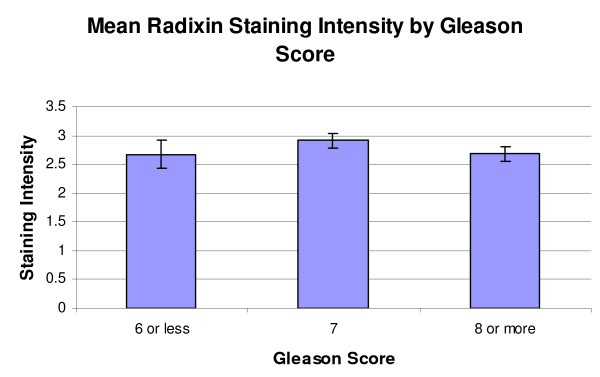
**Mean radixin staining intensity by Gleason score**. Mean radixin staining intensity by Gleason score. No significant differences were seen in this classification (p = 0.938).

**Figure 3:**
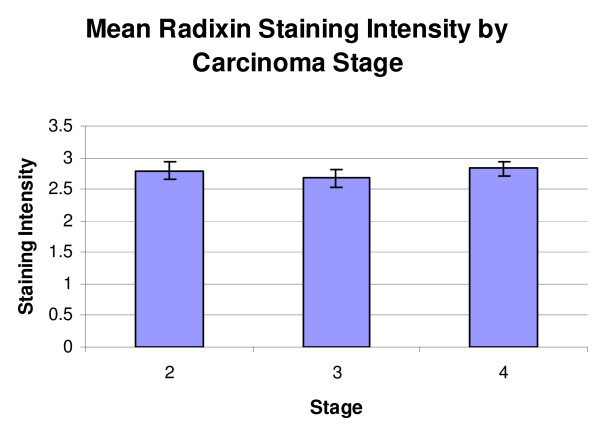
**Mean radixin staining intensity by carcinoma stage**. No significant differences were seen in this classification (p = 0.737).

When classified by Gleason score, the mean staining scores for the radixin-stained TMAs were score 6 or less, 2.67 ± 0.24 (n = 10), score 7, 2.91 ± 0.12 (n = 42), and score 8 or higher, 2.68 ± 0.12 (n = 35) (Figure 2). A resultant One-Way ANOVA showed no significant differences (p = 0.355). When classified by tumor stage, the mean scores for the radixin-stained TMAs were stage 2 or less, 2.79 ± 0.14 (n = 35), stage 3, 2.67 ± 0.14 (n = 26) and stage 4, 2.83 ± 0.11 (n = 25) (Figure 3). A resultant One-Way ANOVA showed no significant differences (p = 0.737).

### Staining Intensities for Moesin

**Figure 4:**
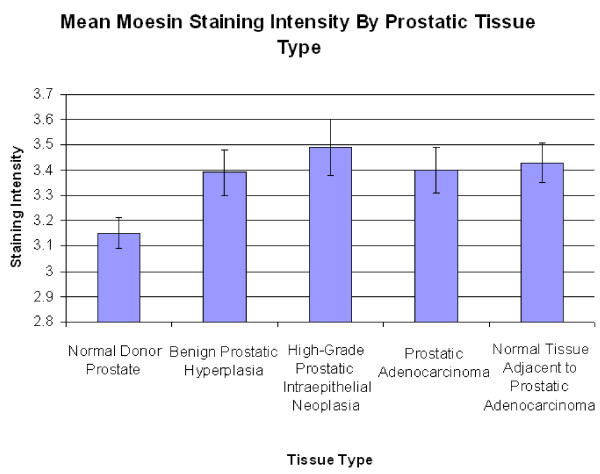
**Mean moesin staining intensity by prostatic tissue type**. No significant differences were seen in this classification (p = 0.152).

The mean staining scores for NDP, BPH, HGPIN, PCa, and NAC in the moesin-stained TMAs were 3.15 ± 0.06, 3.39 ± 0.09, 3.49 + 0.11, 3.40 + 0.09, 3.43 + 0.08 (Figure 4). A Kruskal-Wallis test showed no significant differences between the moesin-stained groups (p = 0.152).

**Figure 5:**
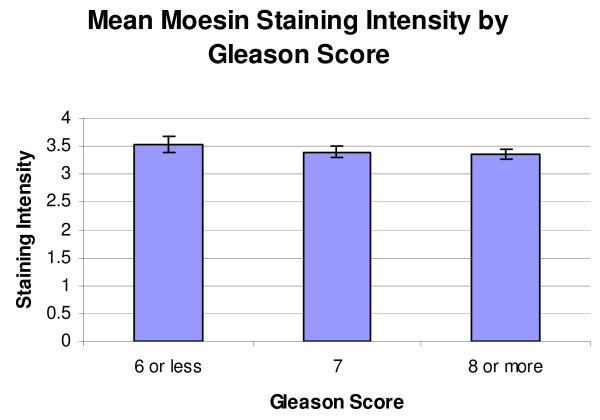
**Mean moesin staining intensity by Gleason score**. No significant differences were seen in this classification (p = 0.719).

**Figure 6:**
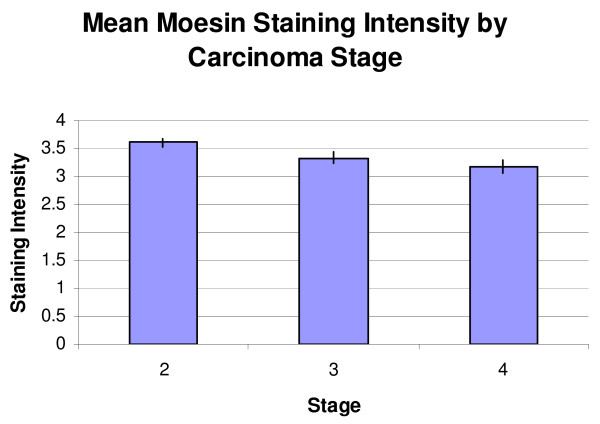
**Mean moesin staining intensity by carcinoma stage**. A significant decrease between Stage 2 and Stage 4 staining was observed (p = 0.003).

When classified by Gleason score, the mean staining scores for the moesin-stained TMAs were score 6 or less, 3.53 ± 0.16, score 7, 3.40 ± 0.09, and score 8 or higher, 3.37 ± 0.08 (Figure 5). A resultant One-Way ANOVA showed no significant differences (p = 0.719). When classified by stage, the mean staining scores for the moesin-stained TMAs were stage 2 or less, 3.62 ± 0.073, stage 3, 3.33 ± 0.10, and stage 4, 3.17 + 0.11 (Figure 6). A resultant One-Way ANOVA (p = 0.003) and subsequent Student-Newman-Keuls test for multiple comparisons showed a significant decrease in Stage 4 staining compared to Stage 2 (p = 0.003).

**Figure 7:**
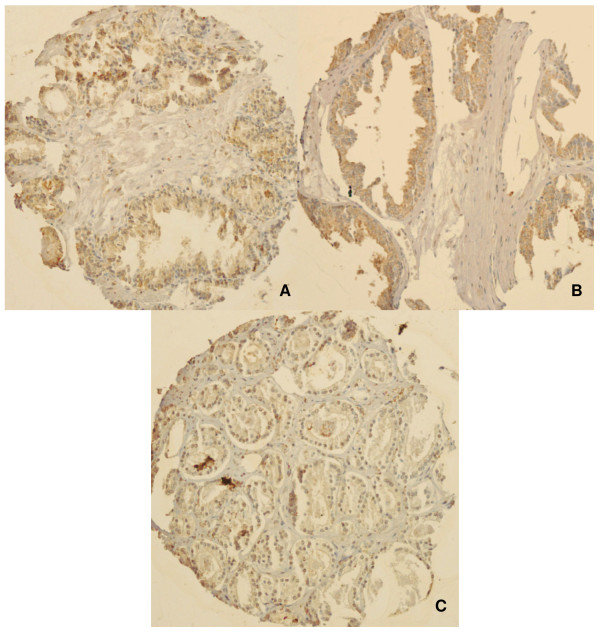
**Photomicrographs of radixin TMA cores**. Representative immunohistochemical staining for radixin in **A) **normal donor prostate **B) **high-grade prostatic intraepithelial neoplasia and **C) **prostatic adenocarcinoma. Radixin staining was diffuse and cytoplasmic. High-grade prostatic intraepithelial neoplasia had a significantly higher staining score than prostatic adenocarcinoma (p < 0.001).

**Figure 8:**
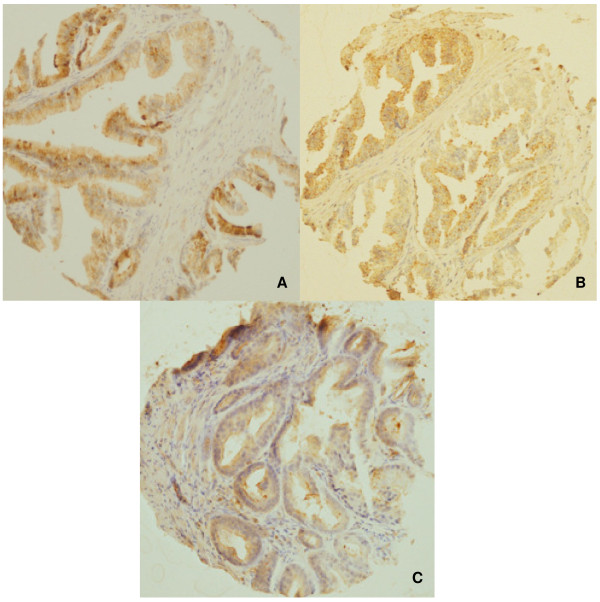
**Photomicrographs of moesin TMA cores**. Representative immunohistochemical staining for moesin is featured in **A**) normal tissue adjacent to prostatic adenocarcinoma **B**) high-grade prostatic intraepithelial neoplasia and **C**) prostatic adenocarcinoma. Moesin staining was also diffuse and cytoplasmic, but was more granular in appearance than radixin. There were no significant differences in staining among the tissue types (p = 0.0152).

Representative photomicrographs of the TMAs are shown in Figures 7 and 8. Radixin staining was diffuse and cytoplasmic. Moesin staining was also diffuse and cytoplasmic, but was more granular in appearance than radixin.

## Discussion

In the radixin-stained specimens, the average intensities were highest in the HGPIN specimens, followed by NDP, and BPH, with the PCa and NAC demonstrating the lowest levels of staining (Figure 1). These absolute differences may support the notion that radixin is down regulated in prostatic adenocarcinoma, given that the lowest intensities were from specimens containing adenocarcinoma, although a One-Way ANOVA with subsequent Student-Newman-Keuls analysis revealed that a significant difference was only observed between HGPIN and NAC (p = 0.001) and HGPIN and PCa (p = <0.001). There are several possible reasons for our finding that specimens of carcinoma have less average staining than HGPIN. This may represent the unique physiological progression of radixin from the pre-neoplastic state to the neoplastic state. Another possibility is that within specimens of HGPIN, there can be a wide spectrum of histologic findings. More specifically, this means that in addition to glands demonstrating HGPIN, there may also be some elements of co-accompanying normal histological architecture, which may assist in imparting a higher staining score on these specimens. This may be true, as previous work has shown radixin to be down regulated in some instances of lungs cancer in comparison to non-tumor lung tissue [12]. The finding that the difference between NDP and PCa specimens was not significant, then, may be a reflection of the sample size of NDPs available for study. As there were fewer NDPs, the natural baseline variability among their expression levels may have had a greater impact in precluding statistical significance despite the absolute staining of NDP being higher than PCa. No significant differences were seen by Gleason score or stage in specimens of prostatic adenocarcinoma (Figure 2 and 3).

In the moesin-stained specimens, the average staining intensities were highest in the HGPIN specimens, followed by NAC, PCa, BPH, and lastly, NDP (Figure 4). No significant differences were seen amongst the groups using a Kruskal-Wallis test (p = 0.152). No significant differences were seen when the adenocarcinoma specimens were stratified by Gleason score. A significant difference was noted between Stage 2 and Stage 4 staining (p = 0.003). The finding that moesin appears to be down regulated from Stage 2 to Stage 4 may seem counterintuitive, as moesin-positive tumors have been shown to demonstrate higher perineural invasion rates in pancreatic adenocarcinoma [17]. However, moesin expression patterns can vary by cancer type, as moesin, like radixin, has been shown to be down regulated in cases of lung cancer [12]. Moreover, it is possible that this finding may reflect a late stage change in tumor physiology.

## Conclusions

These results provide a basis for the characterization of radixin and moesin expression patterns in prostatic adenocarcinoma. More specifically, given that a difference was observed between HGPIN and PCa, this may indicate that radixin has the potential to be a clinically useful biomarker, but larger studies still need to be conducted before any definitive conclusions can be made. Future studies could also look at the expression of radixin in specimens of metastatic prostatic adenocarcinoma in order to determine if radixin is a clinically useful marker to predict the risk of metastasis.

While moesin staining was higher in specimens of PCa than in normal tissue, the staining scores were also higher in HGPIN than they were in PCa, which makes moesin unlikely to be a useful clinical biomarker to diagnose prostate cancer based upon this study. While a significant decrease in moesin staining was noted between Stage 2 and Stage 4 PCas, the actual staining intensities were close in absolute terms (Figure 6). While this difference may reflect a change in physiology in the tissues between stages, additional larger studies will need to be conducted to determine its ability to correlate with stage prior to any clinical implementation.

One proposed model for cell proliferation involving the ERM subfamily of Protein 4.1 indicates roles for both growth promoters and tumor suppressors within the family. In this model, CD44, a glycoprotein, is believed to interact with growth promoting factors, with ERM proteins being phosphorylated and binding to CD44 in the process, leading to a pro-proliferative state[6].

Merlin (moesin-ezrin-radixin-like protein), the product of the neurofibromatosis type 2 (NF2) gene, is another member of the Protein 4.1 superfamily that has an established function as a schwannoma and meningioma tumor suppressor protein, with NF2 mutations also seen in cases of thyroid cancer, mesothelioma, and melanoma [21]. Conversely, it has been proposed to bind CD44 when not phosphorylated, suppressing growth, and is believed to be active in states of high cell densities [5,6]. This is especially interesting when viewed in the light that merlin was shown to be inactivated by constitutive phosphorylation in DU145 line prostate cancer cells [22].

As more studies are conducted in this area, possibly looking at the expression of these markers in metastatic specimens, more definitive roles for the behavior of radixin and moesin in prostate cancer may be discovered, possibly expanding on existing models regarding cell growth and proliferation, and the involvement of members of the Protein 4.1B superfamily in these processes.

## Competing interests

The authors declare that they have no competing interests.

## Authors' contributions

TB assisted in scoring tissue microarrays under the direct supervision of an attending pathologist and drafted the manuscript. UC preformed all statistical calculations. AP conceived of the study, developed and approved the study protocol, approved all tissue microarray scoring, and revised the manuscript. MB also conceived of the study, developed and approved the protocol, and revised the manuscript. All authors read and approved the final manuscript.

## Pre-publication history

The pre-publication history for this paper can be accessed here:

http://www.biomedcentral.com/1472-6890/11/1/prepub
